# Subpleural pulmonary nodule marking with patent blue V dye prior to surgical resection

**DOI:** 10.3389/fonc.2024.1392398

**Published:** 2024-05-21

**Authors:** Vladimír Červeňák, Zdeněk Chovanec, Alena Berková, Petra Cimflová, Martina Kelblová, Ivan Čundrle, Tomáš Hanslík, Jan Resler, Lenka Součková, Natália Jankaničová, Jiří Vaníček

**Affiliations:** ^1^Department of Medical Imaging, St. Anne’s University Hospital Brno and Faculty of Medicine, Masaryk University, Brno, Czechia; ^2^1st Department of Surgery, St. Anne’s University Hospital Brno and Faculty of Medicine, Masaryk University, Brno, Czechia; ^3^Klinik für Neuroradiologie, Universitaetsklinikum Freiburg, Freiburg im Breis, Germany; ^4^Department of Anesthesiology and Intensive Care, St. Anne’s University Hospital Brno and Faculty of Medicine, Masaryk University, Brno, Czechia; ^5^Department of Pharmacology, Masaryk University Faculty of Medicine, Brno, Czechia; ^6^Department of Biostatistics, International Clinical Research Centre of St Anne’s University Hospital Brno, FNUSA, Brno, Czechia

**Keywords:** computed tomography-guided dye labeling, video-assisted thoracoscopic resection, patent blue, pulmonary nodule, lung cancer

## Abstract

**Background and objective:**

Subpleural located pulmonary nodules are perioperatively invisible to the surgeon. Their precise identification is conventionally possible by palpation, but often at the cost of performing a thoracotomy. The aim of the study was to evaluate the success rate and feasibility of the pre-operative CT-guided marking subpleural localized nodule using a mixture of Patent Blue V and an iodine contrast agent prior to the extra-anatomical video-assisted thoracoscopic surgery (VATS) resection in patients for whom the primary anatomical resection in terms of segmentectomy or lobectomy was not indicated.

**Methods:**

The data of consecutive patients with pulmonary nodules located ≤ 30 mm from the parietal pleura, who were indicated for VATS extra-anatomical resection between 2017 to 2023, were retrospectively reviewed and analyzed. All patients indicated for VATS resection underwent color marking of the area with the pulmonary lesion under CT-guided control immediately before the surgery. The primary outcome was the marking success. Morphological lesion characteristics, time from marking to the surgery, procedure related complications, final histology findings and 30day mortality were analyzed. Additionally, we assessed the association of the successful marking and the patient’s smoking history.

**Results:**

A total of 62 lesions were marked. The successful marking was observed in 56/62 (90.3%) patients. The median time from the lesion marking to the beginning of surgery was 75.0 (IQR 65.0-85.0) minutes. The procedure related pneumothorax was observed in 6 (9.7%) patients, intraparenchymal hematoma in 1 (1.6%) patient. No statistically significant association of the depth of the subpleural lesion’s location, occurrence of complications or time from the marking to surgery and the successful marking was observed. The 30day mortality was zero. No association of smoking and successful marking was observed.

**Conclusions:**

The method of marking the subpleural pulmonary lesions under CT-guided control with a mixture of Patent Blue V and iodine contrast agent is a safe and effective method with minimal complications. It provides surgeons the precise visualization of the affected pulmonary parenchyma before the planned extra-anatomical VATS resection.

## 1 Introduction

Pulmonary nodules represent a significant diagnostic challenge within the realms of radiology, pneumo-oncology, and surgery. Their etiology notably varies, encompassing a wide spectrum from benign to malignant lesions including infectious and inflammatory processes, granulomas, primary tumors or metastatic lesions. These nodules are often incidentally detected on computed tomography (CT) of the chest. According to a study by American authors GOOD CA, pulmonary nodules are among the most frequent incidental findings on chest CT, occurring in approximately 50% of smokers over the age of 50 (1). Other studies report the incidence of pulmonary incidentalomas in 15-30% of performed lung CT scans (2).

Technical advances in CT imaging of the lung parenchyma allow improved diagnosis and allow estimation of the oncological potential of these lesions.

There is an array of recommendations and guidelines for the evaluation of pulmonary nodules, which may vary slightly. The recommendations based on the Fleischner classification (3) and BTS guidelines (4) have been recently updated based on the insights from large screening studies (5). They play a crucial role in the assessment of the malignant potential of lesions, risk evaluation, and recommendation of the further management of the pulmonary nodules in daily clinical practice (6).

Small subpleural lesions are challenging for percutaneous transthoracic or transbronchial biopsy, typically resulting in low diagnostic yield (7). Therefore, it is preferred to perform a complete resection of the pathological focus. Video-assisted thoracoscopic surgery (VATS) is often the method of choice (8).

Subpleural lesions are invisible to surgeons during thoracoscopy and their precise location is usually performed by palpation, which can be problematic in minimally invasive approaches due to limited access through a small incision. Subsolid nodules can be difficult to palpate, and pure ground-glass nodules are impalpable (9–11). Therefore, alternative methods of visualization are required. Techniques for visualizing pulmonary nodules are divided into noninvasive, such as localization by ultrasound during surgery (12). Invasive methods involving marking with radiopaque materials such as wires, coils or fiducial markers (13, 14) or staining the resection area with dyes (Patent Blue V, Methylene Blue, indocyanine green) under CT or bronchoscopy guidance (14–18). Dyes are particularly effective in subsolid and pure ground-glass lesions, which are not palpable (8–11). The choice of method depends on facility capabilities and the preferences of radiologists and surgeons. The ideal method should be simple, reliable, and safe.

We have chosen to use color marking with mixture of Patent Blue V and an iodine contrast agent due to its simplicity, safety, excellent results as evidenced in published studies, and its cost-effectiveness. This method allows for precise, reliable localization of lesions.

## 2 Materials and methods

The retrospective study protocol was approved by the ethics committee of the St. Anne´s University Hospital Brno (FNUSA) in the Czech Republic. Patients were informed in detail about their disease, the proposed treatment and the procedure, and based on this information they provided their informed consent prior to the procedure.

### 2.1 Study population

We retrospectively analyzed data from consecutive patients with pulmonary nodules who were indicated for and underwent the extra-anatomical VATS resection with prior marking of these nodules using a mixture of blue dye and contrast agent between 2017 and 2023.

The patients indicated for VATS resection consisted of patients with new incidental findings (incidentalomas), and patients with known oncological disease with newly occurred pulmonary lesions, lesions showing size progression, or lesions with a conspicuous change in their morphological character.

All patients had available baseline CT chest scans that were acquired no longer than 8 weeks prior to the scheduled surgery. Baseline CT chest scans were either conducted at the Department of Medical Imaging at FNUSA or sent to the FNUSA for evaluation from surrounding regional hospitals in the South Moravian region.

### 2.2 Inclusion and exclusion criteria

All patients indicated for VATS resection were individually assessed by institutional multidisciplinary lung tumor board, which included a radiologist, surgeon, oncologist, and pulmonologist. Patients were selected for marking and subsequent surgery after the evaluation of their CT scans, pulmonary examinations (including spirometry and determination of the extent of safe lung resection), oncological assessment, and overall clinical status based on the clinical examinations by an anesthesiologist and internal medicine physician. The evaluation of CT findings was conducted according to the 2017 revised Fleischner Society recommendations and BTS guidelines. A newly diagnosed pulmonary nodule in oncological patients was considered a possible metastasis.

Criteria for VATS resection were solid subpleural nodules larger than 8 mm, subsolid nodules with a solid component larger than 6 mm persisting or progressing in size, conspicuous pure ground-glass nodules larger than 10 mm, and suspected metastatic lesions. When multiple nodules were present, further management was guided by the most conspicuous nodule. All nodules were located within 30 mm of the pleura.

**Figure 1:**
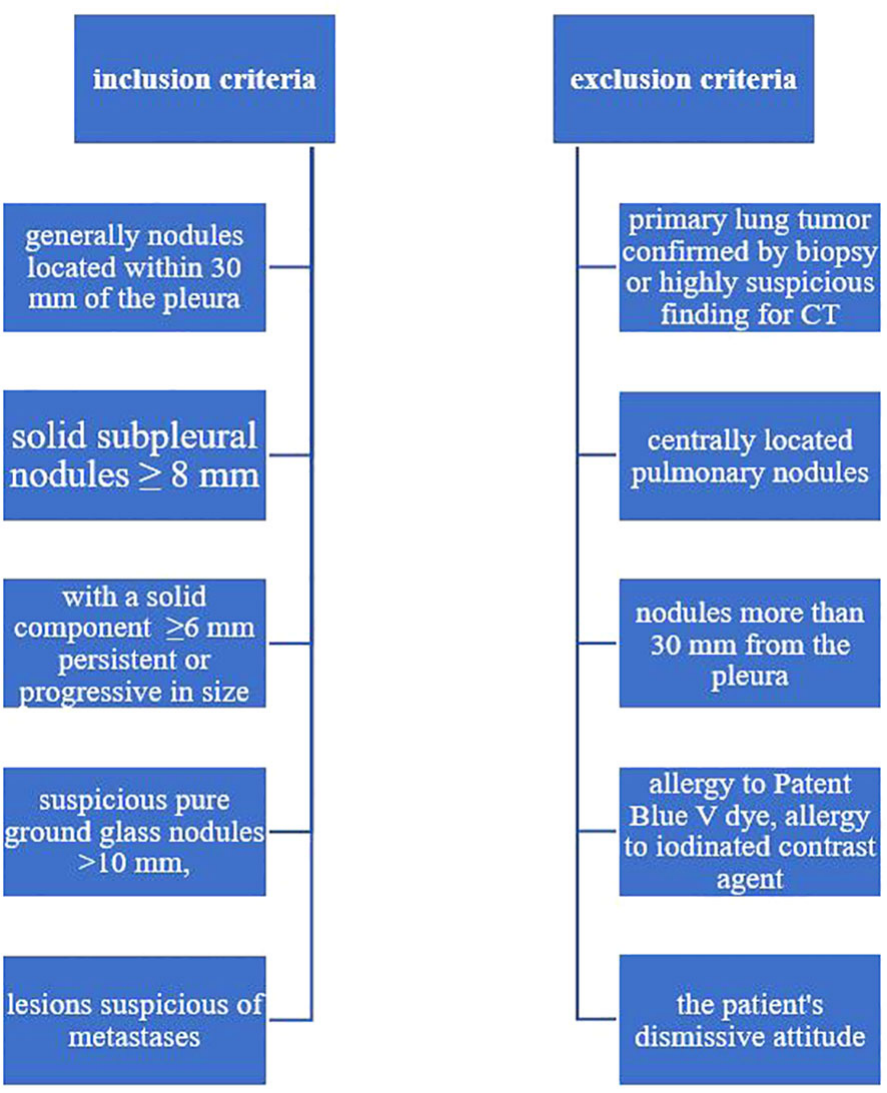
Inclusion and exclusion criteria.

Patients were not indicated for the VATS resection when there was a biopsy-verified primary pulmonary tumor or a highly suspicious finding on CT suggestive of primary pulmonary tumor, centrally located pulmonary nodules or those located more than 30 mm from the pleura. Patients with known allergies to Patent Blue V dye, iodine contrast agents, and those with a negative attitude towards the chosen method were not included in the study. Exclusion criterias were age under 18years, pregnancy, breast feeding, iodine allergy, Patent Blue V allergy, lung nodules obviously crossing parietal pleura. **Figure 1**

### 2.3 Intervention

The marking was performed on the day of the operation and scheduled in such a fashion that the VATS resection of the lesion followed as soon as possible after the marking. On the day of the procedure, a control CT chest was routinely performed for all patients in a supine position to detect any changes.

Unenhanced CT chest exams were performed on Light Speed 64VCT (GE Healthcare) and Brilliance 256 (Phillips Healthcare) machines following a local standardized protocol with slice thickness of 0.625 mm (120 kVp, 100-250 mA). Subsequently, the CT images were reconstructed to slice thicknesses of 5 mm. All images were evaluated both in the lung window (window width 1500 HU, window level -700 HU) and in the mediastinal window (window width 375 HU, window level 50 HU).

Further positioning of the patient (supine, prone and lateral decubitus) on the CT table was adapted to the location of the nodule in the lungs and anatomical conditions, to allow the most precise needle introduction to the nodule. After choosing the optimal and safe puncture path, we marked the puncture site on the patient’s skin using CT coordinate points. In local anesthesia with 1% Mesocain and under aseptic conditions, we introduced the tip of a thin 22G 12 cm long needle to the lesion or its immediate vicinity. The direction and progress of the needle were corrected using the step-by-step method so that the tip safely pointed to the point of interest and passed through the pleura only once. For nodules located near the parietal pleura of the chest wall or near the mediastinal parietal pleura, a trajectory passing through the pleura either perpendicularly or tangentially was chosen. Subsequently, pulmonary tissue was stained by instilling 0.2-0.3 ml of the marking mixture, created by mixing 0.5 ml of iodine contrast agent Omnipaque 350 mgI/mL and 1.5 ml of Patent Blue V in a 2ml syringe. The procedure was completed with a follow-up CT chest to verify the distribution of the marking mixture and to assess any potential complications.

The surgeon was provided with information about the patient’s position on the CT table, the localization of the injected dye in relation to the lesion, and any complications. The patient had a peripheral vein secured throughout the procedure for the administration of corticosteroids and catecholamines in case of an allergic reaction.

**Figure 2:**
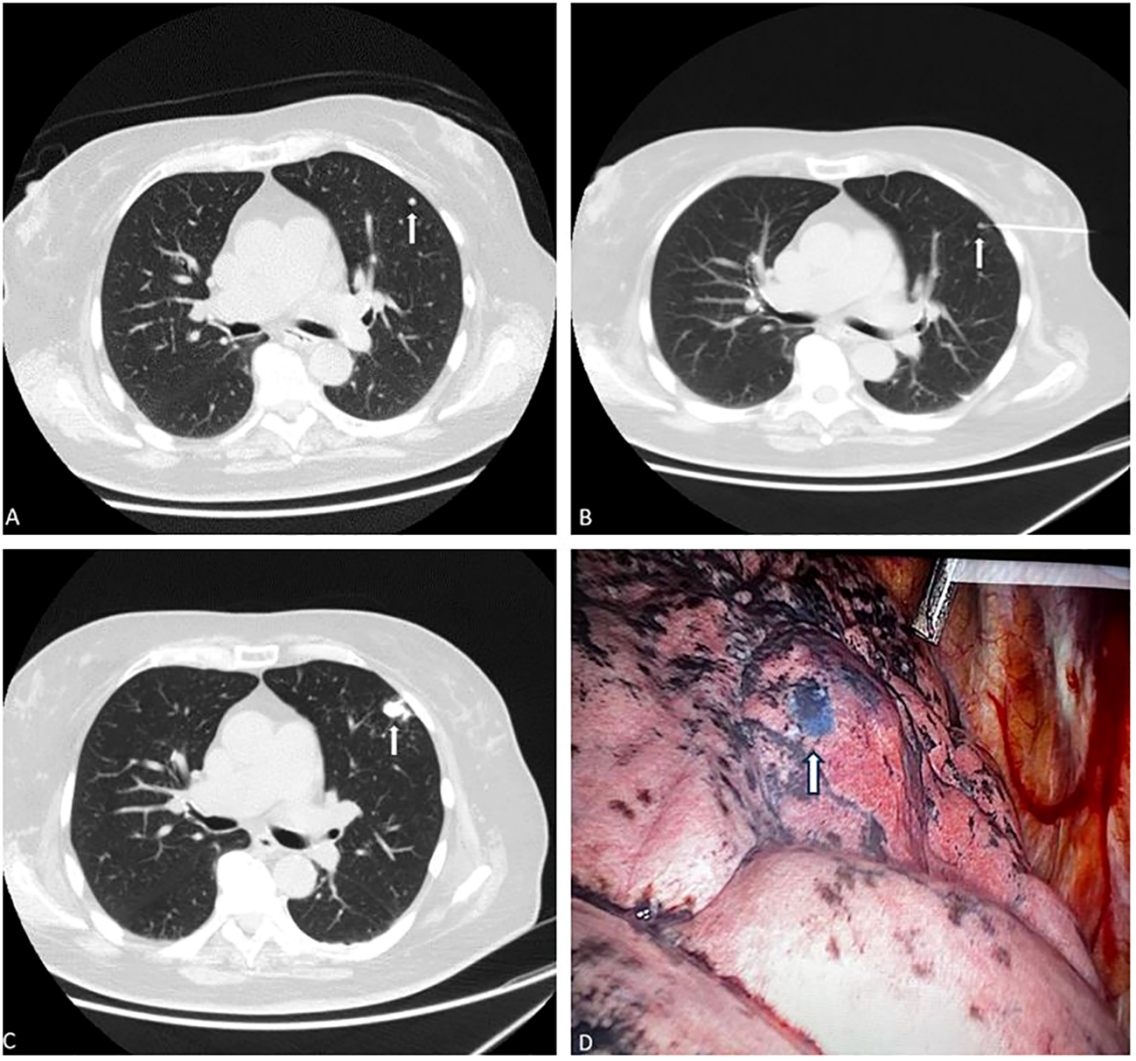
Exemplary case of subpleural lung nodule marking in one patient. Axial CT lung scan demonstrates one solid subpleural nodule in the left upper lobe, **(A, B)** (white arrowhead). **(B)** the needle tip (black/empty arrow) was inserted in the close proximity to the nodule (white arrowhead). **(C)** a marked nodule with a mixture of dye and contrast agent. (it is possible to see the dissolved contrast material due to the planned nodules for resection. **(D)** VATS imaging shows deposition of blue dye in lung tissue (arrow). The location of the dye marking correlates well with preoperative CT scans. The blue marking is clearly visible in the terrain of the anthracotic lung. The lesion histologically corresponded to a malignant melanoma metastasis.

After the marking, patients were transferred to the operating room. Under general anesthesia and selective lung ventilation, VATS resection of the nodule was performed. The marked section was identified by visualizing the blue dye on the surface of the lungs. **Figure 2**.

### 2.4 Outcomes

The primary outcome was the success of the marking.

The criterion for successful marking was defined as clear perioperative visual identification of the blue-marked pulmonary section, enabling safe R0 resection of the lesion during VATS. The failure of marking was defined as inaccurate or impossible perioperative visual identification of the stained pulmonary section. This situation occurred in cases of either complete absence of color marking or when its spread exceeded one pulmonary segment, or in cases of uncertain identification of the marked section within the anthracotic lung tissue.

Other analyzed parameters included the morphological characteristics of the lesion (the number of lesions, their size, location in the lung parenchyma, distance from the pleura), time from marking to the surgical procedure in relation to intraoperative visibility, procedure related complications (pneumothorax, intraparenchymal hematoma) histology findings and 30days mortality. Additionally, we assessed the association of the successful marking and the history of smoking.

### 2.5 Statistical analysis

Statistical analysis was performed using the programming language R (version 4.2.1) in the integrated development environment R studio. Results were considered as statistically significant in a case their p-value was below 0.05. Continuous variables were presented as median and interquartile range (IQR) and categorical variables as numbers with percentages. To test the normality of continuous data Shapiro-Wilk test was performed. Statistical testing of differences between groups consisting of continuous data was performed using Mann-Whitney test as at least one of the compared groups was not normally distributed. Differences between groups consisting of categorical data were statistically tested using Fisher’s test.

## 3 Results

### 3.1 Patient characteristics

The retrospective analysis identified 66 patients. In three patients, complete regression of the lesion was observed on the day of the scheduled procedure, and therefore the intended marking and surgical revision were not performed. One patient on which two markings were performed at once was excluded too. In total 62 (100%) pulmonary lesions were marked. 42 (67.7%) patients had a known oncological history. The cohort included patients aged between 26 and 78 years, with a median age of 57.0 (IQR 49.3-68.0) years. There were 33 (53.2%) women and 29 (46.8%) men. Non-smokers accounted for 44 (71.0%) of the participants, ex-smokers 7 (11.3%), and smokers 11 (17.7%), **Table 1**.

**Table 1:** Patients’ baseline and procedural characteristics.

Category	Data
**Number of Patients**	62
**Patients Age Range, years**	26-78
**Patients Age, median (IQR), years**	57.0 (49.3-68.0)
**Female sex, n (%)**	33 (53.2)
Smoking status, n (%)	
non-smoker	44 (71.0)
ex-smoker	7 (11.3)
smoker	11 (17.7)
**Number of Pulmonary Nodules**	62
Frequency of Pulmonary Nodules, n (%)	
Solitary pulmonary nodule	37 (59.7)
Multiple pulmonary nodules	25 (40.3)
Type of Pulmonary Nodules, n (%)	
Solid pulmonary nodule	60 (96.8)
Subsolid pulmonary nodule	2 (3.2)
Size of Pulmonary Nodules	
Size range, mm	2.0-24.0
Median (IQR), mm	28.5 (6.0-12.0)
Depth of Pulmonary Nodules	
Depth range	1.0-28.0
Median (IQR)	7.0 (4.0-12.8)
Success of Marking, n (%)	
Visible	56 (90.3)
Diffuse	2 (3.2)
Invisible	3 (4.9)
Incorrect location	1 (1.6)
**Unsuccessful Marking, n (%)**	6 (9.7)
**Number of Incidentalomas, n (%)**	30 (48.4)
**Patients with Oncological Diagnosis, n (%)**	42 (67.7)
**Malignant Finding in Resected Tissue, n (%)**	38 (61.3)
Complications, n (%)	
pneumothorax	6 (9.7)
hematoma	1 (1.6)
Time from Marking to Surgery	
Time range, min	28-149
Median (IQR), min	75.0 (IQR 65.0-85.0)

### 3.2 Successful and failed marking

Out of the 62 marked lesions, the marking at the site of the lesion was visible in 56 (90.3%) cases.

Unsuccessful marking was noted in 6 (9.7%) lesions. In 2 (3.2%) cases, the color marking had spread beyond the corresponding pulmonary segment. In 3 (4.9%) cases, the marking was not visible intraoperatively. In 1 (1.6%) marked lesion, the marking was outside of the pathological nodule.

### 3.3 Morphological characteristics of pulmonary nodules and other analyzed parameters

**Figure 3:**
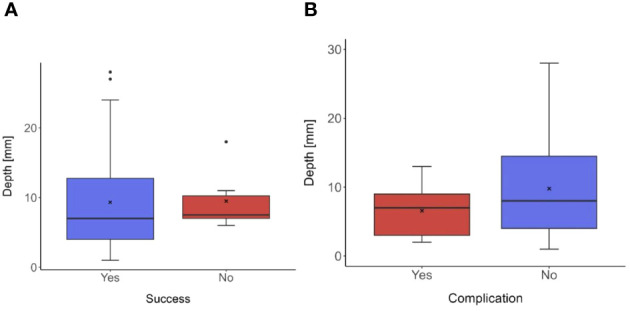
Box plots illustrating the distribution of measured depths in different groups based on the success of marking **(A)** and the occurrence of complications **(B)**. **(A)** The median depth of the pulmonary modules in patients with successful marking was 7.0 mm (IQR 3.8-13.3). In the groups of patients with unsuccessful marking the median of the lesion’s depth was 7.5 mm (IQR 7.0-10.3). Between groups no statistically significant difference was demonstrated, p-value was 0.583. **(B)** The median depth in the group of patients with the occurrence of procedure related complication was 7.0 mm (IQR 3.0-9.0) and in the group without any procedure related complications was 8.0 mm (IQR 4.0-14.5). P-value of the test was 0.332, which indicates, that no statistical significance difference between groups was found.

Thirty-seven patients (59.7%) presented with a solitary pulmonary nodule, and 25 (40.3%) with multiple pulmonary nodules. Solid pulmonary nodules accounted for 60 (96.8%) of the cases, while subsolid pulmonary nodules were 2 (3.2%). There were no pure ground-glass nodules observed in the cohort. The smallest marked nodule was 2.0 mm in size, the largest 24.0 mm, with a median size of the lesions being 8.5 (IQR 6.0-12.0) mm. The most superficially located nodule was at a depth of 1.0 mm from the parietal pleura, and the deepest nodule was at 28.0 mm, with a median depth of 7.0 (IQR 4.0-12.8) mm. No influence of the depth of the lesion’s location on the success of the marking was demonstrated (p = 0.583), **Figure 3A**. There was also no statistically significant difference of depth of the lesion’s location between groups based on occurrence of complications (p = 0.332), **Figure 3B**.

Thirty (48.4%) patients presented with incidentalomas, i.e., incidental findings on CT chest scan. At the time of indication for extra-anatomical VATS resection, 30 (48.4%) patients had one follow-up CT chest, 29 (46.8%) patients had two follow-up CT chest scans, 1 (1.6%) patient had three follow-up CT chest scans, and 1 (1.6%) patient had four CT chest scans available. In one (1.6%) patient there was no information about the number of CT scans.

Forty-two (67.7%) patients had a positive history of known oncological disease. The histology analysis confirmed malignancy in 38/62 (61.3%) patients. In 31 (50.0%) cases, the metastatic process of the pre-existing oncological diagnosis (malignant melanoma, colorectum, lung, kidney, breast) was confirmed. In 6 (9.7%) patients, the primary non-small cell lung carcinoma was diagnosed, and the carcinoid was diagnosed in 1 (1.6%) patient. R0 resection was achieved in all cases.

The procedure related complications occurred in 7 (11.3%) patients. Two types of complications were observed in the cohort: pneumothorax occurred in 6 (9.7%) patients, IP intraparenchymal hematoma occurred in 1 (1.6%) patient. None of these required emergent medical procedures or affected the further course of the surgery.

**Figure 4:**
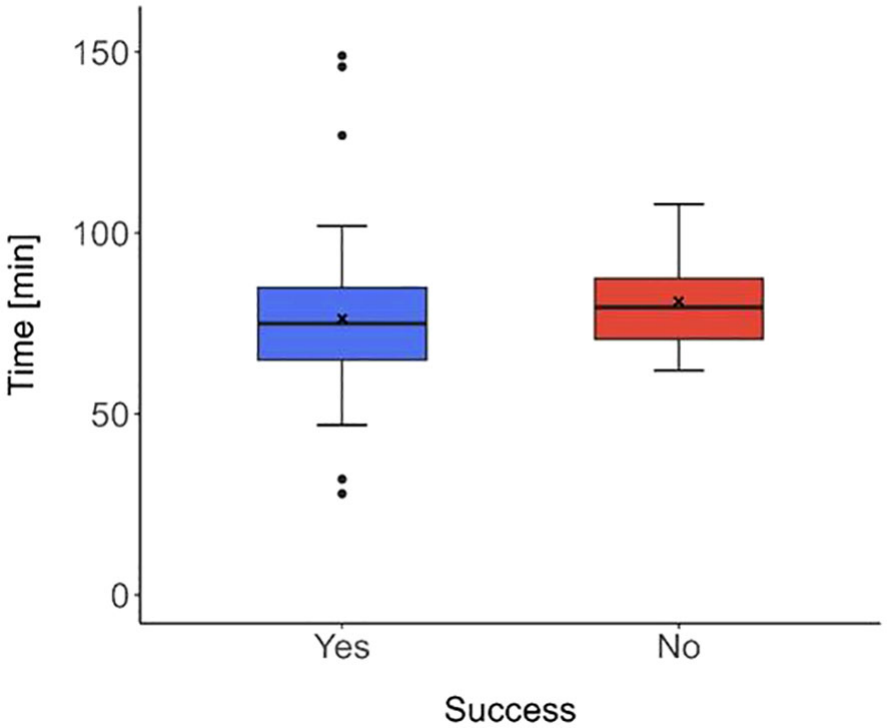
Box plot depicting the distribution of the time from marking to the commencement of surgery concerning the success of marking. The median time in the group of patients with successful marking (blue box plot) was 75.0 min (IQR 65.0-85.0) and in the group, where the marking was not successful (red box plot), the median time was 79.5 min (IQR 70.8-87.5). No significant difference (in the times between groups) was found, with p-value of 0.439.

The median time from the marking of the lesion to the start of the surgery was 75.0 (IQR 65.0-85.0) minutes. The shortest interval was 28 minutes, and the longest was 149 minutes. The time from the end of the marking to the beginning of the surgery did not affect the visibility and the identifiability of the marked pulmonary section, with a p-value of 0.439, **Figure 4**.

**Figure 5:**
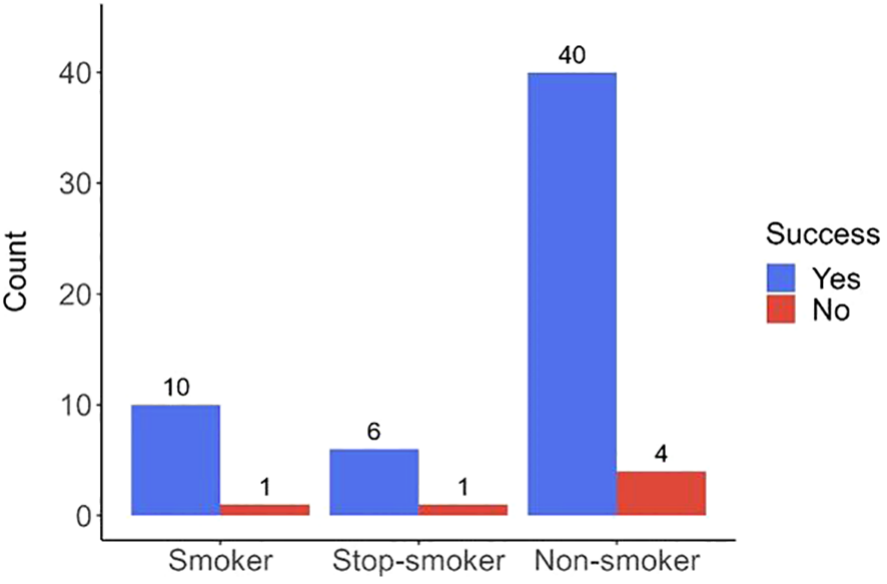
Barplot showing the rates of marking success categorized by patient’s smoking status. The chart displays the number of pulmonary lesions categorized by the smoking status of the patients distinguishing between smokers, ex-smokers, and non-smokers. Blue bars represent the number of lesions successfully marked, while red bars indicate unsuccessful markings. No evidence was found for smoking status to significantly impact the success rate of lesion marking, p-value 0.806.

Additionally, no association of smoking and the visibility of the marking was observed (p = 0.806). Of note, the color marking was well discernible even in the field of anthracotic lung tissue, **Figure 5**.

## 4 Discussion

The development in the management of pulmonary nodules has been influenced by advances in preoperative CT navigation.

Recent studies concur on the key role of these technologies in enhancing the success of subsequent video-assisted thoracoscopy (VATS), especially for solitary or multiple small and subpleural pulmonary nodules, as well as for difficult-to-palpate subsolid lung lesions or impalpable pure ground-glass lesions (11). These methods allow for precise localization of pulmonary nodules (PN), facilitating their resection. Accuracy is particularly crucial when aiming to spare the patient from more burdensome surgical procedures like thoracotomy.

However, each localization method has its advantages and disadvantages. The method of introducing wires or metal coils carries the risk of dislodgement and a higher incidence of complications, such as pneumothorax and bleeding from pulmonary parenchyma (19). The use of metal coils or wires requires intraoperative navigation with X-ray fluoroscopy, increasing the radiation dose to the patient and surgeon (20). Selin Chu et al. report that the dye-marking technique with methylene blue offers shorter procedure and hospitalization times, as well as a safer postoperative course (17). Kleedehn et al. also conclude that the use of methylene blue is as effective as wire introduction but with fewer complications (21). In a study by Yu-Wei Liu using Patent Blue V dye, a marking success rate of 96.6% was achieved (20).

The high efficacy of CT navigation using various techniques, including dyes, metal coils, hooks, or wires, has been demonstrated with technical success mostly exceeding 90% (8, 9, 14, 22). Our study is in keeping with these previously reported high successful rates. The success rate of marking in our cohort reached 90.3%. Failure was recorded in 9.7% of cases. In 3 (4.9%) cases, the marking was not visible intraoperatively. These cases involved lesions located very shallowly under the pleura. Detailed analysis of CT scans revealed that the needle tip did not completely penetrate the pleural layer but only concavely displaced it, resulting in the marking mixture being spilled into the pleural space. In 1 (1.6%) case, the marking was outside the pathological nodule due to the nodule’s close localization under the pulmonary fissure. The marking was performed close to the lesion but above the fissure, therefore the marked section has projected intraoperatively (after dissecting the fissure) into another lobe.

All marking methods using liquid dyes are susceptible to some degree of diffusion. Methylene blue, for example, is known to quickly diffuse in soft tissues, such as during breast surgeries (20). Literature suggests a significant time correlation between the marking of pulmonary lesions and surgery, with the delay advised to be as short as possible (20).

Instillation of lipiodol, the oil-based contrast agent, as a marking medium is notable for its stability and low propensity for diffusion. It can remain in the pulmonary parenchyma for several weeks (23). However, its use requires intraoperative fluoroscopic navigation, and while being water-insoluble, lipiodol represents a potential risk of pulmonary embolism (23).

At our facility, we found the method of marking using a mixture of iodine contrast agent and Patent Blue V dye to be highly effective. The iodine contrast agent clearly visualized the exact distribution of the marking mixture in the pulmonary parenchyma on control scans. According to some authors, to use of this mixture is limited by its rapid diffusion and dispersion in the pulmonary parenchyma (17, 18, 23). On the contrary, Po Chih Chang et al. (20) chose Patent Blue V for their study due to its lower tendency to disperse and lower costs. They reported an interval between marking and surgery ranging from 118 to 520 minutes, with a success rate of up to 96.6%.

In our study, marking dispersion over more than one pulmonary segment was observed in two lesions (3.2%). These patients had a history of pulmonary emphysema and COPD, suggesting a higher susceptibility to dye diffusion. However, given the small number of patients in this group, this cannot be considered significant. In the remaining patients, we did not observe dispersed marking. The longest time delay between marking and surgery was 149 minutes. It is also important to note the potential confusion between methylene blue and Patent Blue V dyes. Though both are blue, they are chemically distinct. Authors reporting dye dispersion mostly work with methylene blue. There is a lack of literature comparing the use of methylene blue and Patent Blue V in the lungs. Patent Blue V is considered to be more stable and may have therefore certain advantages over the methylene blue (23).

Chia-Ying Lin1 et al. (9) report poor visibility of methylene blue markings in the context of anthracotic lungs. Anthracosis is described as a negative factor affecting the visibility of markings in dust-laden lungs. Methylene blue provides a darker, more intense color, which can be problematic in an anthracotic environment. In contrast, Patent Blue V offers a brighter and lighter shade of blue, enhancing its visibility. Yi-Jen Peng et al., in their study of endoscopic dissections, compare methylene blue and Patent Blue V focusing on their dyeing effectiveness and cytotoxicity. Patent Blue V was identified as a more suitable dye for tissues than methylene blue (23). In our study, we did not record any failures of the method related to anthracosis. We were the first to investigate the relationship between the visibility of markings in smokers, ex-smokers, and non-smokers. Statistical analysis didn’t find a significant difference in the visibility of markings between groups of patients based on smoking status, with p-value of 0.806. The color marking was clearly discernible even in anthracotic lungs. However, this finding represents the experience of a single center. The validity of the result is, of course, affected by the small size of the study sample.

Complications associated with these procedures are in general of a minor nature. In our cohort, we recorded 6 (9.7%) cases of procedure related pneumothorax (PNO) and 1 (1.6%) intrapulmonary hemorrhage in connection with the marking procedure. Generally, these procedures can be considered very safe. None of our complications required urgent intervention or prevented subsequent VATS resection. However, there remains a need for careful planning and improvement in intervention techniques. A similar incidence and character of complications are reported in other studies (16–19, 21).

With the use of blue dye, whether methylene blue or Patent Blue V, allergic reactions have been reported, albeit they are extremely rare. These manifest as skin rash, hypotension, and in rare cases, anaphylactic reactions (22). We did not observe any such reactions in our cohort.

Our study has several limitations. First, the results represent the single center experience. Second, the study consists of a relatively small sample, therefore, the validity of the results needs to be interpreted with caution.

The article presents the results of a retrospective analysis of CT-guided marking of subpleural pulmonary lesions prior to planned VATS resection. The results of our study suggest that this method is safe, simple, and well applicable in clinical practice. It provides surgeons with precise visualization of otherwise perioperatively invisible lesions, allowing for the performance of minimally invasive procedures.

## 5 Conclusion

Our research focuses on the technique of marking subpleural lung lesions using a mixture of Patent Blue V dye and iodine contrast agent. This method has demonstrated a high success rate of 90.3%, which is comparable or superior to other published marking techniques, including the use of wires, metal coils, or dyes. Our technique allows the radiologist and surgeon to see the relationship between the color marking and the location of the lesion to be resected immediately after application on CT. This precision in localization contributes significantly to more efficient surgical planning and minimization of errors.

The advantage of this method over the introduction of wires or coils is a lower incidence of complications and significantly lower costs. One vial of Patent Blue V costs less than 3 euros, one spiral costs approximately 40 euros. Studies primarily using Methylene Blue as a dye appear in the literature, whereas studies using Patent Blue V are quite sparse. Our research highlights that Patent Blue V exhibits greater stability to scattering, which is particularly advantageous in anthracotic environments where the color hue of Methylene Blue can be problematic.

Thanks to the incorporation of the contrast fluid into the marking mixture, the surgeon can see the localization of the staining mixture leading to the subpleural nodule and is possible to choose the most optimal resection procedure trajectory.

In this context, it is crucial to emphasize that our study is the first to analyze the stability of Patent Blue V to scattering in detail. These findings contribute to a better understanding and optimization of labeling techniques in pulmonary surgery, which has important implications for surgical practice and patient outcomes.

## Data availability statement

The original contributions presented in the study are included in the article/supplementary material. Further inquiries can be directed to the corresponding author.

## Ethics statement

The studies involving humans were approved by the Ethics Commission of St. Anne’s University Hospital Brno and the Faculty of Medicine (59ML/2021). The studies were conducted in accordance with the local legislation and institutional requirements. The participants provided their written informed consent to participate in this study. Written informed consent was obtained from the individual(s) for the publication of any potentially identifiable images or data included in this article.

## Author contributions

VČ: Investigation, Methodology, Project administration, Validation, Writing – original draft. ZC: Investigation, Methodology, Project administration, Validation, Writing – original draft. AB: Data curation, Writing – review & editing. PC: Writing – review & editing. MK: Writing – review & editing. IČ Writing – review & editing. TH: Writing – review & editing. JR: Writing – review & editing. LS: Writing – review & editing. NJ: Formal Analysis, Writing – review & editing. JV: Writing – review & editing.
